# Alterations in gene expression of *recA* and *umuDC* in antibiotic-resistant *Acinetobacter baumannii*

**DOI:** 10.25122/jml-2022-0358

**Published:** 2023-04

**Authors:** Nada Ali Abdul Ameer, Maysaa Abdul Razzaq Dhahi

**Affiliations:** 1 Bacteriology Laboratory, Al-Imamein Al-Kadhimein Medical City, Baghdad, Iraq; 2 Microbiology Department, College of Medicine, Al-Nahrain University, Baghdad, Iraq

**Keywords:** *Acinetobacter baumannii*, antibiotic resistance, *recA* expression, *umuDC* expression, clinical isolates, ecological isolates, nosocomial infections

## Abstract

*Acinetobacter baumannii* is a critical pathogen with an efficient SOS (Save Our Ship) system that plays a significant role in antibiotic resistance. This prospective descriptive study aimed to investigate the association between expression levels of *recA* and *umuDC* genes, which are critical in SOS pathways, and antibiotic resistance in *A. baumannii*. We analyzed 78 clinical isolates and 31 ecological isolates using the Vitek-2 system for bacterial identification and antibiotic susceptibility testing and confirmed molecular identification of *A. baumannii* by conventional PCR of bla_OXA-51_ and bla_OXA-23_. Quantitative real-time polymerase chain reaction was used to determine gene expression levels of *recA* and *umuDC*. The results showed that in 25 clinical strains, 14/25 strains showed upregulation of *recA*, 7/25 strains exhibited upregulation of both *umuDC* and *recA*, and 1/25 strains showed upregulation of *umuDC*. Of these, 16/25 clinical strains were extensively resistant to antibiotics, except for colistin, and showed upregulation of *recA* and/or *umuDC* gene expression levels. In 6 ecological strains, *recA* showed upregulation in 3/6 strains, while both *recA* and *umuDC* were upregulated in 1/6 strain. In conclusion, high expression levels of *recA* and/or *umuDC* genes in *A. baumannii* complex and *A. baumannii* strains may contribute to increasing resistance to a wide range of antibiotics and may result in the initiation of an extensively drug-resistant (XDR) phenotype.

## INTRODUCTION

*Acinetobacter baumannii* (*A. baumannii*) is a life-threatening pathogen associated with community-acquired and nosocomial infections, mainly pneumonia. In 2017, the World Health Organization declared *A. baumannii* the highest-priority pathogen requiring research and development of new antibiotics. It is one of the six most significant multidrug-resistant (MDR) bacteria in hospitals, with an increasing number of antibiotic-resistant strains, limiting effective treatment options and contributing to higher mortality rates [1,2].

Accurate identification of *Acinetobacter* species remains a challenge for microbiologists, even with the use of commercially available kits such as API 20NE and Vitek 2 systems [3]. One study found that 94.5% of *A. baumannii* strains (163 strains) included in the investigation were carbapenem-nonsusceptible *A. baumannii* (CNSAB), and 90.1% and 52.2% of them were metallo-β-lactamases (MBL) and extended-spectrum β-lactamases (ESBL) producing isolates, respectively [3]. The emergence of antibiotic resistance in bacterial populations is attributed to the activation of the SOS (Save Our Ship) response pathways, which has been shown to result in elevated gene expression and a subsequent increase in mutagenesis. Upon activation, the SOS response elicits an arrest in the cell cycle and a marked increase in the mutation rate [4]. The SOS response pathways in bacteria are regulated by two key genes, *recA* and *umuDC*. These genes are responsible for the formation of DNA Polymerase V, an error-prone polymerase, through the binding of *recA*-mediated cleaved, *umuD*, and *umuC* proteins [5]. In the case of *A. baumannii*, multiple *umuD* and *umuC* proteins play a crucial role in DNA trans-lesion repair and induce mutagenesis, contributing to its antibiotic resistance. These proteins allow the bacteria to replicate DNA across DNA lesions when *recA* is activated [6,7].

The *umuDC* operon plays a crucial role in the temporal regulation of the SOS response. The presence of uncleaved *umuD* and *umuC* proteins in the cell after DNA damage delays the recovery of DNA replication, allowing accurate repair systems to process the damage more effectively [8]. In response to DNA damage, *recA* is activated by binding to single-stranded DNA (ssDNA), which creates a nucleoprotein filament that promotes the self-cleavage of lexA and releases over 50 SOS genes from repression. This response is triggered by the accumulation of intracellular ssDNA, which occurs when DNA polymerase stalls at a lesion while helicase continues to unwind the DNA [9]. The SOS pathway is also pivotal for bacterial pathogenesis. In addition to the two key SOS regulators, lexA and *recA*, other stressors and stress responses can regulate SOS factors. The SOS response plays a critical role in the formation of biofilms, which are highly *reca*lcitrant to antimicrobial agents and can facilitate the formation of persistent cells. Furthermore, the dynamic biofilm environment generates DNA-damaging factors that trigger the SOS response within the biofilm, fueling bacterial variation and diversification [10].

The aim of this study was to investigate the correlation between the expression levels of the key genes in the SOS pathway, *recA* and *umuDC*, and antibiotic resistance in *A. baumannii*.

## MATERIAL AND METHODS

### Study design and sample collection

This prospective, descriptive, cross-sectional study was conducted from December 2020 to September 2021 at Al-Imamein Al-Kadhimein Medical City and Baghdad Medical City in Baghdad, Iraq. A total of 78 clinical isolates were collected from various sources, including sputum (n=35), blood (n=24), urine (n=9), wounds (n=8), and other bodily fluids (n=2), from patients admitted to the hospital. In addition, 31 environmental swabs were collected from various locations within the hospital, including surgical units, intensive care units (ICU), neonatal care units (NICU), and patient wards.

### Identification of *Acinetobacter baumannii* and *Acinetobacter baumannii* complex

An adequate quantity of colonies was taken from the pure culture and suspended in 3 mL of sterile saline. These colonies were then used for identification using the Vitek 2 system

(GNID/AST cards, BioMérieux/France) following the instructions provided by the manufacturer.

For molecular identification, bacterial DNA was extracted from a pellet using the Wizard^®^ Genomic DNA Purification Kit (Promega/USA, Cat. No. A1120) according to manufacturer instructions.

*A. baumannii* and the *A. baumannii* complex were identified by detecting bla_OXA-51_ and bla_OXA-23_ using conventional PCR [11]. A specific primer set was used to detect bla_OXA-51_ and bla_OXA-23_ in the extracted bacterial DNA. A 25 µL PCR master mix was prepared by adding 1X of PCR Buffer (5X) (Promega/USA), 200 µM of dNTPs (Promega/USA), 10 pMol (after serial optimizations) of forward and reverse primers (Alpha/Canada) and 1.5 units of Taq DNA polymerase (Promega/USA). Nuclease-free H_2_O was added to bring the volume to 23 µL. A 2 µL DNA template (50 ng) was added to the reaction tube, and a no-template control (NTC) tube was prepared with all the PCR master mix components but with nuclease-free H_2_O (2 µL) instead of DNA. The PCR reaction tubes were transferred to a thermal cycler (Eppendorf, Germany) programmed to run at 94°C for 5 min (1X), 30 cycles of 94°C for 1 min, 55°C for 30 sec (bla_OXA-51_) or 55°C for 1 min (bla_OXA-23_) (after serial optimizations), 72°C for 1.5 min, and a final extension of 72°C for 7 min. To confirm the presence of bla_OXA-51_ and bla_OXA-23_ genes, the PCR products were separated by electrophoresis on 1.5% agarose gel, and the presence of a band with a molecular size of 353 bp and 501 bp, respectively, indicated a positive result for each gene.

### Antibiotic susceptibility testing

An appropriate number of colonies were transferred from pure culture and suspended in 3 mL of sterile saline using a sterile cotton swab. The turbidity of the bacterial suspension was corrected to the 0.5 MacFarlandreagent using a visible spectrophotometer (DensiChek TM Plus). The bacterial suspension was inoculated onto the identification card of the Vitek 2 system (GNID/AST cards, BioMérieux/France, Cat no., A 222). The bacterial suspension was placed in a test and fixed into a particular rack, while the identification cards (IDGN card for bacterial identification) and AST cards (for antibiotic susceptibility) were fixed into contiguous slots. The Vitek 2 system enables the analysis of test reactions utilizing heterogeneous visible wavelengths. Each test reaction is scanned every 15 minutes during incubation to measure either turbidity or the colored effect of substrate metabolism. To avoid false readings caused by small bubbles, a specific algorithm is applied. The results become visible after 6 hours of incubation.

### Quantification Real Time-PCR

Total RNA was extracted from bacterial pellet using the SV Total RNA Isolation System (Promega/USA, Cat. No. Z3100), according to the manufacturer’s instructions. Messenger RNA (mRNA) was then reverse transcribed to complementary DNA (cDNA) using the GoTaq^®^ 2-Step RT-qPCR System (Promega/USA, Cat. No. A6010). The concentration and purity of extracted DNA, RNA, and cDNA were measured using a Nano-drop apparatus (LanYuXuan, China).

The expression levels of *recA, umuDC*, and 16rRNA (housekeeping gene) were estimated in the cDNA of selected *A. baumannii* and *A. baumannii* complex isolates (39/78), based on molecular identification, using the GoTaq^®^ 2-Step RT-qPCR System (Promega/USA, Cat. No. A6010) following the manufacturer's instructions. The primer set for amplification was selected according to Bustin et al. [12]. Briefly, a 20µl reaction mixture was prepared per reaction, containing 1X of 2X GoTaq^®^qPCR Master Mix, forward and reverse primers (10pMol for *recA*,5 pMol for *umuDC* and 2 pMol for 16rRNA, after optimization) and Nuclease-Free H_2_O added up to 15 µl. The cDNA template concentration was standardized to 150 ng/5µl for all samples. Subsequently, 5µl of diluted cDNA was added to each RT-Q-PCR master mix tube. A no-template control tube was prepared by adding all RT-qPCR master mix components with 5µl of nuclease-free H_2_O instead of cDNA. Reaction tubes were placed in a real-time thermal cycler (Mic, Australia) and programmed to run at 95°C for 2 min (1x) and 35 cycles of 95°C for 15 sec and 61°C for 1 min. The relative expression level of the studied genes was calculated using the fold change 2^−ΔΔCT^ method [13] as follows:

ΔCt = Ct of target gene (*recA* or *umuDC*) – average Ct value of the housekeeping gene (16S RNA).

ΔΔCt = (ΔCt of target gene (*recA* or *umuDC*) - ΔCt of control group )

Note: The control group was *A. baumannii* and *A. baumannii* complex strains sensitive to all antibiotic categories.

Fold change = 2^−ΔΔCt^

The results of gene expression analysis were interpreted as follows: a value of 0 represents no change in expression, a value greater than 0 indicates gene upregulation and a value less than 0 indicates gene downregulation.

### Statistical analysis

Data were collected, summarized, analyzed, and presented using the Statistical Package for Social Sciences (SPSS) version 23 and Microsoft Office Excel 2010. Categorical variables were presented as frequencies and percentages, while normally distributed continuous variables were expressed as mean (± standard deviation) and range after evaluating the normality distribution using the Kolmogorov-Smirnov test. Various statistical tests were used, including the chi-square test, to assess the association between two categorical variables, with Yates correction applied when the expected count was less than 5 in more than 20% of cells. Spearman correlation was used to evaluate the correlation between two numeric variables, with the results presented as correlation coefficient (r) and level of significance (P). The Kappa agreement statistic was used to assess the degree of concordance between the molecular and Vitek 2 system tests. Sensitivity, specificity, positive predictive value (PPV), and negative predictive value (NPV) were calculated using standard formulas. The significance level was considered at a P-value equal to or less than 0.05. A p-value equal to or less than 0.01 was considered highly significant.

## RESULTS

### Demographic distribution

This study analyzed 78 clinical samples and 31 environmental swab samples. Table 1 shows the socio-demographics of the patients, including age and sex, with a male-to-female ratio of 1.23:1. The mean age was 36.5 ± 23.5 years, ranging from neonates (less than 1-year-old) to 82 years. The results showed that most infected patients were in the age group of 31-40 years.

**Table 1:** Distribution of patients according to age and sex.

Age (year)	Sex		Total
	Male	Female	
**>1**	6 (54.5%)	5 (45.4%)	11 (14.1%)
**1–10**	4 (80%)	1 (20%)	5 (6.41%)
**11–20**	3 (42.8%)	4 (57%)	7 (8.9%)
**21–30**	0 (0%)	2 (100%)	2 (2.56%)
**31–40**	9 (47.3%)	10 (52.6%)	19 (24.3%)
**41–50**	6 (66.6%)	3 (33.3%)	9 (11.5%)
**51–60**	5 (50%)	5 (50%)	10 (12.8%)
**61–70**	8 (72.7%)	3 (27.2%)	11 (14.1%)
**71–80**	1 (33.3%)	2 (66.6)	3 (3.84%)
**>80**	1 (100%)	0 (0%)	1 (1.2%)
**Total**	43 (55.1 %)	35 (44.9%)	78 (%)

### Identification of *Acinetobacter baumannii* and *Acinetobacter baumannii* complex

**Figure 1:**
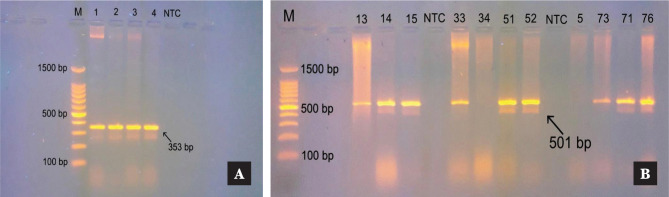
Agarose gel electrophoresis of PCR-amplified products of blaOXA-51 and bla_OXA-23_ genes in *A. baumannii*. (A): Lane 1-4: amplified products of bla_OXA-51_ (353bp) form different strains. M: molecular size ladder of 100bp. NTC: no template control. (B): Lane 13,14,15,33,51,52,71,73,76: amplified products of bla_OXA-23_ (501 bp) from different strains. Lane 5 and 34: no amplified products of bla_OXA-23_. M: molecular size ladder of 100bp. NTC: no template control. Electrophoresis was done on a 1.5% agarose gel at 5V/cm for 90 min.

The results of bacterial identification using the Vitek 2 system showed that 17/78 (21.7%) clinical isolates were *A. baumannii*, and 61/78 (78.2%) were *A. baumannii* complex. The molecular identification of 78 clinical isolates and 31 ecological isolates showed that 29.4% (23/78) of clinical isolates were identified as *A. baumannii* using bla_OXA-51_ and bla_OXA-23_, while 70.5% (55/78) were identified as *A. baumannii* complex. Seven additional clinical isolates were identified as negative for bla_OXA-51_ and bla_OXA-23_ but as 6 *A. baumannii* complex and 1 *A. baumannii* using Vitek 2 system. Of the 31 ecological isolates, 5 (16%) were identified as *A. baumannii* complex using Vitek 2, and 6 (19.3%) were identified as *A. baumannii* complex using molecular identification. The results of PCR amplification of bla_OXA-51_ and bla_OXA-23_ are displayed in Figure 1 A–B.

### Antibiotic susceptibility

The antibiotic susceptibility of 78 clinical isolates of *A. baumannii* and *A. baumannii* complex was assessed using the Vitek 2 system (see Supplement 1). The antibiogram showed that the highest resistance was observed for cefoxitin (87.1%) and cefepime(87.1%), with 68 out of 78 isolates (16 *A. baumannii* and 52 *A. baumannii* complex) resistant. Conversely, colistin showed the lowest resistance rate, with only 1 out of 78 (1.2%) isolates being resistant. The 7 ecological isolates showed complete resistance to cefoxitin, cefepime, and ceftazidem, while only 1 of these isolates showed resistance to colistin (Supplement 1).

### Bacterial identification using Vitek 2 system and molecular identification using conventional PCR

We evaluated 78 clinical isolates from samples of patients with various infections using Vitek 2 system and molecular identification. Results indicated that 70.51% of the isolates (55 out of 78) were identified as *A. baumannii* complex through molecular analysis, while 46 isolates were identified as *A. baumannii* complex and 9 as *A. baumannii* by Vitek 2 system. There was a slight agreement between the results obtained from Vitek 2 system and that from molecular identification, with an accuracy of 69.23%. The comparison between the results obtained from these two identification techniques was not statically significant (p=0.307) (Table 2).

**Table 2:** Comparison of clinical isolate identification using Vitek 2 and molecular techniques.

Vitek 2 system	Molecular technique		Total
	*A. baumannii*	*A. baumannii* complex	
***A. baumannii***	8	9	17
***A. baumannii* complex**	15	46	61
**Total**	23	55	78
**Statistic**	for *A. baumannii*	for *A. baumannii* complex	-
**Kappa agreement**	0.20 ^†^	0.20 ^†^	-
**Sensitivity %**	83.64	34.78	-
**Specificity %**	34.78	83.64	-
**PPV %**	75.41	47.06	-
**NPV %**	47.06	75.41	-
**Accuracy %**	69.23	69.23	-
**p-value**	0.307 Mc NS		-

† – slight agreement; Mc – McNemar test; NS – not significant.PPV – positive predictive value; NPV – negative value NPV; p – p-value

There was no significant correlation between the results obtained from the identification of ecological isolates using the Vitek 2 system and those obtained using molecular techniques (p = 1.000), as shown in Table 3.

**Table 3:** Comparison of ecological isolate identification using Vitek-2 and molecular techniques.

Vitek 2 system	Molecular technique		Total
	*A. baumannii*	A. baumanii complex	
***A. baumannii***	0	2	2
***A. baumannii* complex**	1	4	5
**Total**	1	6	7
**Statistic**	for *A. baumannii*	for *A. baumannii* complex	-
**Kappa agreement**	-0.24 ^†^	-0.24 ^†^	-
**Sensitivity %**	66.67	0.00	-
**Specificity %**	0.00	66.67	-
**PPV %**	80.00	0.00	-
**NPV %**	0.00	80.00	-
**Accuracy %**	57.14	57.14	-
**p-value**	1.000 Mc NS		-

† – No agreement; Mc – McNemar test; NS – not significant; PPV – positive predictive value; NPV – negative value NPV; p – p-value.

### Relationship between clinical sample types and antibiotic susceptibility

The clinical isolates obtained from sputum samples exhibited a high level of antibiotic resistance. Specifically, 50% of the 52 *A. baumannii* complex strains isolated from sputum samples were resistant to cefoxitin, 54% to ceftazidime, and 59% to ciprofloxacin. While there was no significant association between sample type and resistance to cefoxitin (p=0.086), we found a significant association between sample type and resistance to both ceftazidime and ciprofloxacin (p=0.042 and p=0.001, respectively) as reported in Supplement 2. We also observed that 43 out of 78 (55.1%) *A. baumannii* complex strains and 11 out of 78 (14.1%) *A. baumannii* strains were sensitive to colistin. The highest percentage of *A. baumannii* complex strains that were sensitive to all antibiotics were found in sputum samples (19/43, 44.1%), followed by blood samples (17/43, 39.5%). This finding was statistically highly significant (p-value <0.002) (Supplement 2).

### Quantification of recA and umuDC expression levels using quantitative Real-Time PCR

In order to assess the gene expression levels of *recA* and *umuDC*, 39 strains were analyzed based on their molecular identification and antibiotic susceptibility patterns. However, only 31 (79.4%) strains, including 25 clinical and 6 ecological isolates, produced interpretable results for *recA* and/or *umuDC* expression. The remaining 8 (25.8%) strains failed to produce any results even after repeating the experiment and were thus excluded from the analysis.

The results of gene expression levels in clinical strains of *A. baumannii* showed that *recA* was upregulated in 3/25 (12%) strains, and both *recA* and *umuDC* were upregulated in 2/25 (8%) strains. In one strain (4%), expression levels of both *recA* and *umuDC* were unchanged. In clinical strains of *A. baumannii* complex, expression of *recA* only was upregulated in 11/25 (44%) strains, *umuDC* only was upregulated in 1/25 (4%) strain, and both *recA* and *umuDC* were upregulated in 5/25 (20%) strains. In 3/25 (12%) strains of *A. baumannii* complex, expression of *recA* was unchanged, while expression of *umuDC* was unchanged in 4/25 (20%) strains. The results of gene expression levels of *recA* and *umuDC* in ecological strains of *A. baumannii* and *A. baumannii* complex showed that 50% of the strains had upregulated *recA* expression, while only one strain of *A. baumannii* complex had upregulated expression of both *recA* and *umuDC*. No correlation was found between the gene expression levels and molecular identification of *A. baumannii* and *A. baumannii* complex, with p-values of 1.000 and 0.362, respectively. These findings are presented in Tables 4, 5, and Supplement 3.

**Table 4:** Association between molecular identification and gene expression level of recA in *Acinetobacter baumannii* and *Acinetobacter* complex strains.

RecA	*Acinetobacter baumannii*		*Acinetobacter baumannii* complex		P
	N	%	N	%	
**No change**	1	14.3	5	20.8	1.000 YNS
**Upregulation**	6	85.7	19	79.2	
**Total**	7	100.0	24	100.0	

Y – Yates correction test; NS – not significant; p – p-value.

**Table 5:** Association between molecular identification and gene expression level of umuDC in *Acinetobacter baumannii* and *Acinetobacter* complex strains.

UmuDC	*Acinetobacter baumannii*		*Acinetobacter baumannii* complex		P
	N	%	N	%	
**No change**	4	66.7	3	30.0	0.362 Y
**Upregulation**	2	33.3	7	70.0	NS
**Total**	6	100.0	10	100.0	-

Y – Yates correction test; NS – not significant; p – p-value.

### The relationship between the expression levels of recA and umuDC in *Acinetobacter baumannii* and *Acinetobacter* complex strains

There was no correlation between the gene expression levels of *recA* and *umuDC* in *A. baumannii* and *A. baumannii* complex (p = 1.000) (Table 6).

**Table 6:** Correlation between gene expression levels of recA and umuDC in *Acinetobacter baumannii* and *Acinetobacter* complex.

umuDC	recA				P
	No change		Up regulation		
	N	%	N	%	
**No change**	1	50.0	6	42.9	1.000 Y NS
**Upregulation**	1	50.0	8	57.1	
**Total**	2	100.0	14	100.0	

Y – Yates correction test; NS – not significant; p – p-value

### The relationship between sample type and gene expression levels of recA and umuDC

The relationship between the source of isolation and gene expression levels of *recA* and/or *umuDC* was investigated for 25 clinical strains. Blood samples had elevated expression levels of *recA* and *umuDC*. Specifically, the expression of *recA* was elevated in 2/10 *A. baumannii* strains and 3/10 *A. baumannii* complex strains, while both *recA* and *umuDC* were elevated in 3/10 *A. baumannii* strains (30%), as seen in Table 7.

**Table 7:** The relationship between clinical sample type and gene expression of recA.

recA	Sample type						P
	Sputum	Blood	Urine	Wound	Endotracheal	Total	
**No change**	1	2	0	1	0	4	0.918 C ^†^ NS
**Up regulation**	5	8	3	4	1	21	
**Total**	6	10	3	5	1	25	

C – chi-square test; ^†^ – more than 20 % of cells have an expected count of less than 5; NS – not significant; p –p-value.

The gene expression level of *umuDC* was upregulated in only 1/7 (14.2%) *A. baumannii* complex strain, while both *umuDC* and *recA* were upregulated in 2/7 strains of *A. baumannii* and 1/7 strain *A. baumannii* complex (Table 8). However, there was no significant relationship between the sample types and gene expression levels of *recA* and *umuDC* (p=0.918 and p=0.692, respectively).

**Table 8:** The relationship between clinical sample type and gene expression of umuDC.

umuDC	Sample type						P
	Sputum	Blood	Urine	Wound	Endotracheal	Total	
**No change**	1	3	0	1	1	6	0.692 C ^†^ NS
**Up regulation**	2	4	1	1	0	8	
**Total**	3	7	1	2	1	14	

C – chi-square test; ^†^ – more than 20 % of cells have an expected count of less than 5; NS – not significant; p –p-value

### The relationship between gene expression levels of recA and umuDC in strains isolated from clinical and ecological samples

The expression levels of *recA* and *umuDC* were analyzed in 25 clinical and 6 environmental strains of *A. baumannii* and *A. baumannii* complex. The results indicated that 21/25 (84%) of the clinical strains had upregulated expression levels of *recA*, while 4/6 (66.6%) of the ecological strains had upregulated levels. However, there was no statistically significant correlation between the gene expression levels of *recA* in clinical and ecological strains (p = 0.375), as shown in Table 9.

**Table 9:** Correlation between the gene expression level of recA in clinical strains and ecological strains.

recA	Clinical strains		Ecological strains		P
	N	%	N	%	
**No change**	4	16.0	2	33.3	0.375 Y NS
**Up regulation**	21	84.0	4	66.7	
**Total**	25	100.0	6	100.0	

Y – Yates correction test; NS – not significant; N – number of strains.

The gene expression levels of *umuDC* showed an upregulation in 8 out of 14 (57%) clinical strains and 1 out of 2 (50%) ecological strains. The correlation between gene expression levels of *umuDC* in clinical and ecological strains was not statistically significant (p-value = 1.000,) as shown in Table 10.

**Table 10:** Correlation between gene expression levels of umuDC in clinical strains and ecological strains.

umuDC	Patients		Ecological		P
	N	%	N	%	
**No change**	6	42.9	1	50.0	1.000 YNS
**Up regulation**	8	57.1	1	50.0	
**Total**	14	100.0	2	100.0	

Y – Yates correction test; NS – not significant; N – number of strains.

### The relationship between antibiotic susceptibility and gene expression levels of recA and umuDC

There was no correlation between the expression levels of *recA* and *umuDC* genes and the susceptibility of *A. baumannii* and *A. baumannii* complex strains to antibiotics, as determined by quantifying gene expression levels in 25 clinical strains and 6 environmental strains that had previously undergone antibiotic susceptibility testing. The statistical analysis of these results was not significant, as seen in Table 11.

**Table 11:** Relationship between antibiotic susceptibility using Vitek 2 system and gene expression level of recA and umuDC.

Antibiotic	recA		umuDC	
	r	P	R	P
**TIC**	0.197	0.392	0.316	0.407
**TIC-CLV**	0.198	0.390	0.316	0.407
**PIP**	0.042	0.827	0.218	0.417
**PIP-TAZ**	0.035	0.856	0.198	0.461
**CAZ**	0.033	0.859	0.218	0.417
**CEF**	0.033	0.859	0.218	0.417
**CXN**	0.196	0.299	0.101	0.710
**CEX**	0.215	0.246	0.101	0.710
**ETN**	0.237	0.243	0.150	0.609
**IMP**	-0.096	0.608	-0.036	0.894
**MER**	-0.026	0.910	0.561	0.073
**AK**	-0.270	0.174	0.444	0.128
**GM**	-0.280	0.142	0.198	0.461
**TOB**	-0.141	0.542	0.168	0.643
**MNO**	-0.321	0.366	0.000	1.000
**COL**	-0.204	0.351	0.516	0.104
**TIG**	-0.051	0.802	0.124	0.674
**CIP**	-0.036	0.849	0.051	0.851
**LEV**	-0.267	0.255	-0.314	0.377
**TRI**	0.054	0.773	0.163	0.547

* r – Spearman rank coefficient; p – p-value; TIC – Ticarcillin; TIC-CLV – ticarcillin-clavulanic acid; PIP – piperacillin; PIP-TAZ – piperacillin-tazobactam; CAZ – ceftazidime; CEF – cefepime; CXN – ceftriaxone; CEX – cefoxitin; ETN – Ertapenem; IMP – imipenem; MER – meropenem; MNO – minocycline; AK – Amikacin; GM – Gentamicin; TOB – Tobramycin; TIG – tigecycline; TRI – Trimethoprim-sulfamethoxazole; LEV – Levofloxacin; CIP – ciprofloxacin; COL – Colistin.

## DISCUSSION

### Identification of *A. baumannii* and *A. baumannii* complex

Due to the high prevalence of *A. baumannii* infections in hospital settings, particularly in ICUs and NICUs, accurate and timely diagnosis is crucial for effective infection control. Specific identification methods, such as molecular techniques like PCR, are essential for precise and rapid diagnosis, as they provide high sensitivity and specificity. Furthermore, early detection and appropriate treatment within 6-36 hours are critical for ICU patients, as delayed or inadequate treatment may result in increased morbidity and mortality rates [14].

The molecular identification of *A. baumannii* and *A. baumannii* complex in clinical and environmental isolates using bla_OXA-51_ and bla_OXA-23_ demonstrated a 69.23% agreement with the Vitek 2 system identification results, as presented in Table 2. However, the observed discrepancy may be attributed to the superior accuracy of molecular identification, considered the gold standard technique for precise species identification of these bacterial strains, compared to the Vitek 2 system. Several studies have investigated the prevalence of blaOXA genes in *A. baumannii* and *A. baumannii* complex isolates. In an Iraqi study conducted in 2020, bla_OXA-51_ was found in all clinical isolates tested (54/54, 100%), while bla_OXA-23_ was the predominant gene in *A. baumannii* isolates (49/54, 90.74%) [15]. Similarly, another Iraqi study in 2021 reported that bla_OXA-51_ was present in all isolates tested (22/22, 100%), while bla_OXA-23_ was detected in 18/22 (81%) isolates [16]. A study conducted in Jordan in 2022 on 622 clinical isolates of *A. baumannii* confirmed by both Vitek 2 and molecular identification showed that all isolates were positive for bla_OXA-51_ (100%), and 98.5% of isolates were positive for bla_OXA-23_ [17].

A study conducted in Iran in 2022 found that among 85 *A. baumannii* isolates (53 from various surfaces of the hospital environment and 32 from burn patients), 38.5% of hospitalized patients with burn wounds and 22.1% of surfaces, including burn units (15.6%) and intensive care units (52.4%), were positive for *A. baumannii*. The antibiotic susceptibility testing using the disk diffusion method revealed that all isolates from burn patients were resistant to imipenem [18].

*A. baumannii* is known for its ability to cause outbreaks due to its multidrug resistance (MDR) and tolerance to desiccation, which facilitates its persistence in hospital environments. Factors that contribute to *A. baumannii* infection include procedures such as surgery, central catheter placement, tracheostomy, mechanical ventilation, and enteral feeding, as well as treatment with third-generation cephalosporins, fluoroquinolones, and carbapenems [19]. However, contamination with transient or normal flora can occur during the collection of clinical samples, making it difficult to distinguish between contamination and confirmed infection. This can result in false-positive culture results, leading to longer patient stays, increased antibiotic use, and higher preclinical investigation costs [20].

### Antibiotic susceptibility test

In the current study, a significant increase in resistant isolates of *A. baumannii* and *A. baumannii* complex was observed, as indicated in Supplement 1. This growth may be due to the widespread use of antibiotics such as carbapenems, quinolones, and third-generation beta-lactams, which are the most effective antibiotics. *A. baumannii* can acquire and spread drug resistance genes through various mechanisms, such as plasmids, integrons, and transposons, which are interchangeable genetic elements that play a crucial role in the transfer of antibiotic resistance genes [21]. A study in Duhok, Iraq conducted in 2019 found that 6.8% of the *A. baumannii* isolates (41/603) obtained from clinical samples were resistant to most antibiotics tested [22]. The only effective antimicrobial agent was colistin. Another study in China (2021) showed that 81.2% of *A. baumannii* strains were resistant to carbapenem and 100% to cephalosporins, with over 70% resistant to quinolones and aminoglycosides [23].

### Quantification of the gene expression levels of recA and umuDC in *A. baumannii* and *A. baumannii* complex isolated from clinical and environmental samples

This study found no significant correlation between gene expression levels of *recA* and *umuDC* in both clinical and environmental strains of *A. baumannii* and *A. baumannii* complex, as demonstrated in Tables 7 and 8. However, the limited sample size may have affected the statistical power of the analysis. A study conducted in Taiwan (2015) confirmed the presence of the *recA* gene in all *Acinetobacter* species using a multiplex PCR-based assay [24]. Another study found that the wild type of *A. baumannii* ATCC 17978 requires regulation of *recA* for DNA damage transcriptome and has a specialized role for the *UmuD*Ab repressor. They discovered that 152 genes in the standard strain were dependent on *recA*. The 152 gene-induced transcriptomes consisted of two DNA damage-induced regulons: 123 genes regulated by *recA* alone and 27 genes regulated by both *recA* and *umuD*Ab [25].

In this study, there was no correlation between sample types and gene expression levels of *recA* and *umuDC*, which may be due to the small sample size. High expression of *umuDC* and *recA* was observed in strains isolated from blood, sputum, and urine samples. This could be due to various factors such as contaminated conditions, the patient's immune status, the nature and location of the infection, and the use of invasive instruments like endotracheal tubes and cardiovascular catheters, which may increase the likelihood of bacteria being resistant to antibiotics.

Strain 66, isolated from the blood sample of a 76-year-old male ICU patient with sepsis bacteremia, showed a 5.46-fold increase in *recA* expression. Strain 21, isolated from the blood sample of a 3-day-old male NICU patient with bacteremia, had a 36.25-fold upregulation in *umuDC* expression. Strain 37, isolated from the urine sample of an 18-year-old female outpatient with a urinary infection, showed an 85.6-fold increase in *recA* expression and a 0.57-fold increase in *umuDC* expression. Strain 69, isolated from the endotracheal sample of a 74-year-old male ICU patient with pulmonary infection, showed a 6.77-fold upregulation in *recA* expression.

Failure to implement antimicrobial stewardship programs to improve the appropriate use of antibiotics and infection control significantly contributed to the transmission of resistant strains of bacteria like *Acinetobacter spp*., especially among ICU patients [26].

### Correlation between recA and umuDC gene expression levels and antibiotic susceptibility of *A. baumannii* and *A. baumannii* complex strains

The strains that showed upregulation in gene expression levels of *recA* and/or *umuDC* were completely resistant to meropenem and completely sensitive to colistin. The sensitivity to colistin may be due to its rapid bactericidal effect through interactions with lipids, causing a rupture in the outer membrane, leading to changes in cell permeability, leakage of cellular content, and cell death. It could also be because of a reduced ability to repair damaged DNA [27]. A study in Spain (2015) found that different classes of antimicrobial agents used to treat *A. baumannii* infections (such as meropenem, colistin, ciprofloxacin, and tetracycline) can induce mutagenesis in this pathogen. The study found that ciprofloxacin and tetracycline induce mutagenesis through the SOS-mediated mechanism, while colistin and meropenem, commonly used in clinical therapy, do not induce mutagenesis [28]. In 2021, an Indian study on the *A. baumannii* strain ATCC 17978 analyzed the transcriptome after exposure to high concentrations of ciprofloxacin and found that genes involved in the SOS response (*recA, umuDc*, and *ddrR*) were upregulated [29].

An increase in bacterial antibiotic resistance is largely due to the acquisition of new mutations through DNA damage repair. In response to DNA damage, cells activate the DNA damage response (DDR), which increases DNA damage tolerance. This is achieved by employing Y-family DNA polymerases that can bypass lesions. However, these DNA polymerases have low accuracy and can result in replication errors, some of which lead to antibiotic resistance. In *A. baumannii*, multiple genes encode DNA Pol V, which are organized as operons like *umuDC* and unlinked genes [30].

## Conclusion

The study highlights the prevalence of *A. baumannii* complex strains among ICU patients and their high resistance to multiple antibiotics, including ESBL and fluoroquinolones. The upregulation of *recA* and *umuDC* gene expression levels in *A. baumannii* complex strains may contribute to their increased resistance to a wide range of antibiotics and the potential initiation of the XDR phenotype.

## Supplementary Material


