# Supplementary material for: Alterations in gene expression of recA and umuDC in antibiotic-resistant Acinetobacter baumannii
Source: J Med Life. 2023 Apr;16(4):531–9. doi: 10.25122/jml-2022-0358 (PMC10251391; doi:10.25122/jml-2022-0358)
Supplement: Supplementary file 1 [file JMedLife-16-531-s001.pdf]

Supplemental data file.

Supplement 1. Antibiotic susceptibility test using Vitek-2 system.

Antibiotic	Results of susceptibility test	Total No. of clinical isolates	Total No. of ecological isolates	AB	ABC
TIC	R	27	5	6	21
	I	1		0	1
	S	5	2	1	4
TIC-CLV	R	27	5	6	21
	S	6	2	1	5
PIP	R	56	7	13	43
	I	1	NI	0	1
	S	18	NI	3	15
PIP-TAZ	R	58	7	13	45
	I	2	NI	0	2
	S	17	NI	3	14
CAZ	R	63	7	15	48
	S	15	NI	2	13
CEF	R	68	7	16	52
	S	9	NI	1	8
CXN	R	66	6	16	50
	S	8	1	1	7
CEX	R	68	7	16	52
	S	9	NI	1	8
ETN	R	46	7	13	33
	I	2	NI	0	2
	S	10	NI	1	9
IMP	R	56	6	15	41
	I	4	NI	0	4
	S	18	1	2	16
MER	R	42	2	11	31
	I	3	NI	0	3
	S	15	2	2	13
AK	R	49	6	11	38
	S	22	1	5	17
GM	R	49	2	11	38
	I	2	NI	2	0
	S	22	2	4	18
TOB	R	28	6	2	26
	I	1	NI	0	1
	S	13	1	4	9
NET	R	8	NI	1	7
	S	1		0	1
TRT	R	13	NI	1	12
	I	2	NI	0	2
	S	7	NI	1	6
MNO	R	5	NI	2	3
	I	7	NI	1	6
	S	16	NI	3	13
PLO	R	6	NI	3	3

Supplement 1. Continued.

Antibiotic	Results of susceptibility test	Total No. of clinical isolates	Total No. of ecological isolates	AB	ABC
**LEV**	R	36	6	10	26
	I	1	NI	0	1
	S	15	1	2	13
**CIP**	R	59	5	15	44
	S	18	2	2	16
**COL**	R	1	1	0	1
	I	4	NI	1	3
	S	54	6	11	43
**TIG**	R	2	2	1	1
	I	4	NI	2	2
	S	61	5	14	47
**RIF**	1	1	NI	0	1
	S	5	NI	2	3
**TRI**	1	54	6	13	41
	S	23	1	4	19

TIC – Ticarcillin; TIC-CLV – Ticarcillin-clavulanic acid; PIP – piperacillin; PIP-TAZ – piperacillin-tazobactam; CAZ – ceftazidime; CEF – cefepime; CXN – ceftriaxone; CEX – cefoxitin; ETN – Ertapenem; IMP – imipenem; MER – meropenem; MNO – minocycline; AK – Amikacin; GM – Gentamicin; TOB – Tobramycin; NET – Netelmycin; TRT – Tetracyclin; PLO – pleofloxaci; TIG – tigecycline; RIF – rifampicin; TRI – Trimethoprim/sulfamethoxazole; LEV – Levofloxacin; CIP – ciprofloxacin; COL – Colistin; AB – *Acinetobacter baumannii*; ABC – *Acinetobacter baumannii* complex; NI – Not identified.

Supplement 2. Relationship between sample types and antibiotic susceptibility.

Vitek-2			Clinical Sample							P
			Blood	Endotracheal	Fluid	Sputum	Urine	Wound	Total	
**AB**	TIC	R	2	NI	0	2	1	1	6	0.670 C
		S	1	NI	0	0	0	0	1	
**ABC**	TIC	R	5	1	1	8	3	3	21	0.292 C NS
		I	1	0	0	0	0	0	1	
		S	0	0	0	0	3	1	4	
**AB**	TIC-CLV	R	2	0	0	2	1	1	6	0.670 C NS
		S	1	NI		0	0	0	1	
**ABC**	TIC-CLV	R	5	1	1	8	3	3	21	0.292 C NS
		S	1	0	0	0	3	1	5	
**AB**	PIP	R	3	NI	0	8	1	1	13	0.104 C NS
		S	3	NI	0	0	0	0	3	
**ABC**	PIP	R	10	1	1	23	4	4	43	0.291 C NS
		I	1	0	0	0	0	0	1	
		S	7	0	0	2	4	2	15	
**AB**	PIP-TAZ	R	3	NI	0	8	1	1	13	0.104 C NS
		S	3	NI	0	0	0	0	3	
**ABC**	PIP-TAZ	R	10	1	1	25	4	4	45	0143 C NS
		I	2	0	0	0	0	0	2	
		S	6	0	0	2	4	2	14	
**AB**	CAZ	R	4	NI		8	1	2	15	0.245 C NS
		S	2	NI		0	0	0	2	

Supplement 2. Continued.

Vitek-2			Clinical Sample							P
			Blood	Endotracheal	Fluid	Sputum	Urine	Wound	Total	
**ABC**	CAZ	R	12	1	1	26	4	4	48	0.042 C*
		S	6	0	0	1	4	2	13	
**AB**	CEF	R	5	NI		8	1	2	16	0.583 C NS
		S	1			0	0	0	1	
**ABC**	CEF	R	15	1	1	25	5	5	52	0.252 C NS
		S	3	0	0	1	3	1	8	
**AB**	CXN	R	5	NI	0	8	1	2	16	0.583 C NS
		S	1	NI	0	0	0	0	1	
**ABC**	CXN	R	14	1	1	24	5	5	50	0.212 C NS
		S	3	0	0	0	3	1	7	
**AB**	CEX	R	5	NI	0	8	1	2	16	0.583 C NS
		S	1	NI	0	0	0	0	1	
**ABC**	CEX	R	14	1	1	26	5	5	52	0.086 C NS
		S	4	0	0	0	3	1	8	
**AB**	ETN	R	4	NI	0	8		1	13	0.379 C NS
		S	1	NI	0	0		0	1	
**ABC**	ETN	R	8	1	1	17	3	3	33	0.165 C NS
		I	2	0	0	0	0	0	2	
		S	3	0	0	1	4	1	9	
**AB**	IMP	R	4	NI	0	8	1	2	15	0.245 C NS
		S	2		0	0	0	0	2	
**ABC**	IMP	R	10	1	1	20	4	5	41	0.770 C NS
		I	2	0	0	2	0	0	4	
		S	6	0	0	5	4	1	16	
**AB**	MER	R	3	NI	0	5	1	2	11	0.286 C NS
		S	2	NI	0	0	0	0	2	
**ABC**	MER	R	6	1	1	16	3	4	31	0.169 C NS
		I	2	0	0	1	0	0	3	
		S	5	0	0	1	5	2	13	
**AB**	AK	R	2	NI	0	7	0	2	11	0.096 C NS
		S	3	NI	0	1	1	0	5	
**AB**	GM	R	3	NI	0	6	0	2	11	0.487 C NS
		I	1	NI	0	1	0	0	2	
		S	2	NI	0	1	1	0	4	
**ABC**	GM	R	8	1	1	21	3	4	38	0.021 C *
		S	10	0	0	2	4	2	18	
**AB**	TOB	R	1	NI	0	1	0	NI	2	0.269 C NS
		S	3	0	0	0	1	NI	4	
**ABC**	TOB	R	6	NI	0	15	2	3	26	0.079 C NS
		I	1	0	0	0	0	0	1	
		S	5	0	0	0	2	2	9	
**AB**	NET	R		NI	0	1		NI	1	
**ABC**	NET	R	1	NI	0	6	0	NI	7	0.018 C *
		S	0		0	0	1		1	
**AB**	TRT	R	0	NI	0	1		NI	1	0.157 C NS
		S	1	NI	0	0		NI	1	

Supplement 2. Continued.

Vitek-2			Clinical Sample							P
			Blood	Endotracheal	Fluid	Sputum	Urine	Wound	Total	
**ABC**	TRT	R	4	NI	0	6	0	2	12	0.353 C NS
		I	1	NI	0	1	0	0	2	
		S	1		0	3	2	0	6	
**AB**	MNO	R	0	NI	0	1	1	NI	2	0.384 C NS
		I	1	NI	0	0	0	NI	1	
		S	2	NI	0	1	0	NI	3	
**ABC**	MNO	R	0	0	0	2	1	0	3	0.687 C NS
		I	1	0	0	2	1	2	6	
		S	5	1	1	3	2	1	13	
**ABC**	PLO	R	1	NI	0	2		NI	3	
**AB**	LEV	R	2	NI	0	6		2	10	0.091 C NS
		S	2	NI	0	0		0	2	
**ABC**	LEV	R	5	1	1	2	1	NI		0.023 C NS
		I	0	1	0	0	1			
		S	8	1	3	0	13			
**AB**	CIP	R	4	NI	0	8	1	2	15	0.245 C NS
		S	2	NI	0	0	0	0	2	
**ABC**	CIP	R	7	1	1	26	5	4	44	0.001 C **
		S	11	0	0	0	3	2	16	
**AB**	COL	I	1	NI	0	0	0	0	1	0.536 C NS
		S	3	NI	0	6	1	1	11	
**ABC**	COL	R	0	NI	0	0	0	1	1	0.002 C **
		I	0	NI	0	1	0	2	3	
		S	17	NI	0	19	5	2	43	
**AB**	TIG	R	1	NI	0	0	0	0	1	0.854 C NS
		I	1	NI	0	1	0	0	2	
		S	4	NI	0	7	1	2	14	
**ABC**	TIG	R	0	0	0	1	0	0	1	0.802 C NS
		I	1	0	0	0	0	1	2	
		S	12	1	1	23	6	4	47	
**AB**	RIF	S	1	NI	0	1		NI	2	
**ABC**	RIF	R	0	NI	0	1	0	NI	1	0.513 C NS
		S	1	NI	0	1	1	NI	3	
**AB**	TRI	R	4	NI	0	7	0	2	13	0.193 C NS
		S	2	NI	0	1	1	0	4	
**ABC not identified**	TRI	R	11	0	1	22	3	4	41	0.082 C NS
		S	7	1	0	4	5	2	19	
			24	1	1	35	9	8	78	

C – chi-square test; † – more than 20 % of cells have an expected count of less than 5; NS – not significant; NI – Not identified; TIC – Ticarcillin; TIC-CLV – Ticarcillin-clavulanic acid; PIP – piperacillin; PIP-TAZ – piperacillin-tazobactam; CAZ – ceftazidime; CEF – cefepime; CXN – ceftriaxone; CEX – cefoxitin; ETN – Ertapenem; imp – imipenem; MER – meropenem; MNO – minocycline; AK – Amikacin; GM – Gentamicin; TOB – Tobramycin; NET – Netelmycin; TRT – Tetracyclin; PLO – pleofloxaci; TIG – tigecycline; RIF – rifampicin; TRI – Trimethoprim/sulfamethoxazole; LEV – Levofloxacin; CIP – ciprofloxacin; COL – Colistin.

Supplement 3. Gene expression level quantification of *recA* and *umuDC* in *A. baumanni* and *A. baumannii* complex using quantitative Real-Time PCR.

Isolate ID	Molecular identification		Bacterial identification	Antibiotic susceptibility Phenotype	Gene expression level			
	blaOXA- 51	blaOXA-23			Fold change (*umuDC*)	Gene expression level of (*umuDC*)	Fold change (*recA*)	Gene expression level of (*recA*)
**49 C**	POS	NEG	ABC	XDR		NI	1.58	upregulated
**70 C**	POS	POS	AB	XDR	0.007	No change	0	No change
**30 C**	POS	NEG	ABC	XDR	0.14	upregulated	6.02	upregulated
**44 C**	POS	NEG	ABC	MDR		NI	0.053	No change
**46 C**	POS	NEG	ABC	NDR	0.02	No change	0.16	upregulated
**51 C**	POS	POS	AB	MDR	0	No change	1.494	upregulated
**6 C**	POS	NEG	ABC	XDR	NI	NI	0.132	upregulated
**45 C**	POS	NEG	AB	NDR	NI	NI	1	upregulated
**28 C**	POS	POS	AB	NDR	10.26	UP	0.66	UP
**3 C**	POS	NEG	ABC	MDR	NI	NI	0.34	upregulated
**22 C**	POS	POS	AB	XDR	NI	NI	0.32	upregulated
**17 C**	POS	NEG	ABC	XDR	7.41	upregulated	3.91	upregulated
**16 C**	POS	POS	AB	XDR	24.08	upregulated	0.75	upregulated
**23 C**	POS	NEG	ABC	XDR	NI	NI	0.57	upregulated
**21 C**	POS	NEG	ABC	XDR	36.25	upregulated	0.39	upregulated
**20 C**	POS	NEG	ABC	MDR	2.2	upregulated	0.05	No change
**67 C**	POS	POS	AB	XDR	0.006	No change	1.65	upregulated
**66 C**	POS	NEG	ABC	XDR	NI	NI	5.46	upregulated
**26 C**	POS	NEG	ABC	NDR	NI	NI	0.1	upregulated
**60 C**	POS	NEG	ABC	XDR	0.47	upregulated	0.6	upregulated
**39 C**	POS	NEG	ABC	XDR	NI	NI	1.67	upregulated
**37 C**	POS	NEG	ABC	NDR	0.57	upregulated	85.6	upregulated
**62 C**	POS	NEG	ABC	XDR	0.01	No change	1	upregulated
**24 C**	POS	NEG	ABC	XDR	NI	NI	0.02	No change
**69 C**	POS	NEG	ABC	XDR	0.006	No change	6.77	upregulated
**47 E**	POS	NEG	ABC	XDR	0.17	upregulated	0.93	upregulated
**35 E**	POS	NEG	ABC	XDR	NI	NI	0.01	No change
**32 E**	POS	POS	AB	XDR	0.09	No change	0.25	upregulated
**34 E**	POS	NEG	ABC	MDR	NI	NI	0.0042	No change
**33 E**	POS	NEG	ABC	MDR	NI	NI	0.2	upregulated
**52 E**	POS	NEG	ABC	XDR	NI	NI	0.16	upregulated

AB – *Acinetobacter baumannii*; ABC – *Acinetobacter baumannii* complex; MDR – multidrug-resistant extensively drug-resistant; POS – positive; NEG – negative; Fold change =$2^{-\Delta\Delta CT}$; C – clinical strain; E – ecological strains; NI – Not identified.
